# Employees' preference analysis on lean six sigma program coaching attributes using a conjoint analysis approach

**DOI:** 10.1016/j.heliyon.2023.e17846

**Published:** 2023-07-03

**Authors:** Anna Luisa C. Guevarra, Yogi Tri Prasetyo, Ardvin Kester S. Ong, Klint Allen Mariñas

**Affiliations:** a School of Industrial Engineering and Engineering Management, Mapúa University, 658 Muralla St., Intramuros, Manila 1002, Philippines; b School of Graduate Studies, Mapúa University, Manila, Philippines. 658 Muralla St., Intramuros, Manila 1002, Philippines; c International Bachelor Program in Engineering, Yuan Ze University, 135 Yuan-Tung Road, Chung-Li 32003, Taiwan; d Department of Industrial Engineering and Management, Yuan Ze University, 135 Yuan-Tung Road, Chung-Li 32003, Taiwan; e Department of Industrial and Systems Engineering, Chung Yuan Christian University, Taoyuan 320, Taiwan

**Keywords:** Lean, Six sigma, Project coaching, Conjoint analysis, Employee preference

## Abstract

The purpose of this study was to determine the preference of employees for Lean Six Sigma project coaching attributes. Conjoint analysis with an orthogonal design was utilized and six attributes were considered: coaching style, frequency of coaching sessions, duration of coaching sessions, the turn-around time of feedback, documentation review, and mock defense. In addition, 4 different projects were also evaluated: Quick Win project, Yellow Belt project, Green Belt project, and Black Belt project. The results showed that for Quick Win projects, employees consider conducting a mock defense as the highest preference, followed by having a documentation review, and a democratic coaching style. For Yellow Belt projects, employees considered conducting a mock defense as the highest preference, followed by having a documentation review, and a weekly coaching session. For Green Belt projects, employees consider having a documentation review as the highest preference, followed by a transactional coaching style, and conducting a mock defense. Lastly, for Black Belt projects, employees consider having a documentation review as the highest preference, followed by conducting a mock defense, and a 1-week turn-around time for feedback. The results of this study will help companies to implement and to sustain better employee-oriented LSS programs.

## 1 Introduction

Lean and Six Sigma have started as two separate streams of thought for operations improvement [1]. As defined by the American Society for Quality [2], Lean is a set of management practices that are used to improve efficiency and effectiveness by eliminating waste. Its core principle is to reduce and eliminate non-value-adding activities. On the other hand, ASQ defined Six Sigma as a method that improves the capability of an organization's business processes which results in an increase in performance and a decrease in process variation. The results of applying Six Sigma help lead to defect reduction and improvement in profits. With the benefits of Six Sigma, McFarren [3] stated that many organizations worldwide have implemented this methodology and have achieved improvements in their market share, customer satisfaction, and performance of products and services.

Lameijer et al. [1] further explained that it is only in recent years that the Lean and Six Sigma methodologies are applied and studied as one. Lean Six Sigma (LSS) is defined by ASQ as a data-driven philosophy of improvement that values defect prevention rather than defect detection [4]. LSS creates a competitive advantage by reducing variation, waste, and cycle time, while promoting the use of work standardization and flow. It is deployed in organizations as a program or a collection and execution of LSS projects [1]. These LSS projects typically follow the rigorous DMAIC approach. According to ASQ, DMAIC or Define, Measure, Analyze, Improve, and Control is a five-phase data-driven quality strategy used to improve processes. The DMAIC process defines the problem, measures process performance, analyzes the process, improves process performance, and controls the improved process by implementing long-lasting solutions. While DMAIC is not the only methodology in use, it is the most widely adopted and recognized.

DMAIC projects can further be classified into the following “belts”. Antony and Karaminas [5] discussed that at the project level, there are Master Black Belts, Black Belts, Green Belts, and Yellow Belts. These employees conduct projects and implement improvements. Yellow Belts participate as a project team member for improvement projects. Green Belts lead improvement projects and support data collection and analysis for Black Belt projects. Black Belts lead projects that show extensive use of systems and tools to showcase the DMAIC methodology. Master Black Belts completed several Black Belt projects, train and coach Black Belts and Green Belts, and act as the internal LSS consultant [6].

**Fig. 1:**
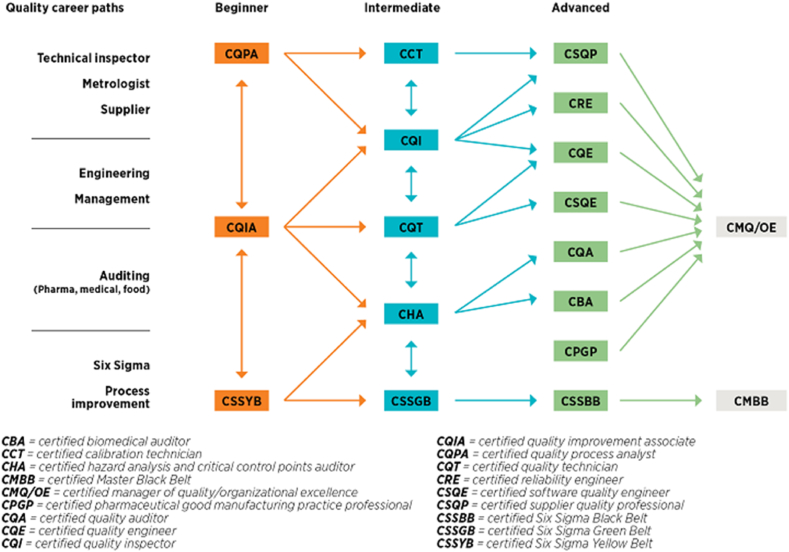
Common Six Sigma Belt Career Pathways [6].

As seen in Fig. 1, the career path and progression of an employee in Six Sigma starts with Yellow Belt, then Green Belt, Black Belt, and finally Master Black Belt. For an employee to be certified under a respective belt, one must undergo an LSS training, pass the certification exam, and complete a Six Sigma project.

Another type of LSS project is called a Quick Win project. Rodrigues et al. [7] defined Quick Win as a rapid improvement in the production abilities of an organization within a short period of time. In addition, GoLeanSixSigma [8] stated that a Quick Win project typically implements a simple solution to a known issue. Given that Quick Win projects require little analysis, less experienced Six Sigma professionals will work on them. LSS projects, both DMAIC and Quick Win, contribute significantly to the improvement of an organization's operations [7,9]. This is on the premise that the implementation of LSS projects has been successful.

Successful LSS projects can contribute to a company's quest to align with Industry 4.0. Sodhi [10] mentioned that employees who are trained in Lean Six Sigma and have experience with continuous improvement can take an active role in preparing their companies to adapt new technologies in their operations. Established quality processes empower dynamic and efficient analyses of vast amounts of data. While there are benefits for a successful implementation of LSS projects, there are challenges too.

Gijo et al. [11] mentioned that one of the hurdles in implementing Six Sigma in the organization is the improper selection of belts, with employees switching over jobs frequently because of market demands. Furthermore, it is said that the belts should have a strong will to improve. Gay [12] added that apart from belt selection, a crucial factor of success is coaching. It was mentioned that coaches play an important role in guiding inexperienced Green Belts in implementing the DMAIC methodology. When inexperienced Green Belts are not provided with sufficient coaching and support, they most likely will make mistakes and these mistakes might lead to project failure. Proper coaching plays a role in preventing this undesirable outcome.

Hagen [13] explained that the idea of utilizing coaching during the process of implementing a Six Sigma project is not a new one. The job of a Black Belt is to manage a process improvement project via the facilitation, training and coaching project of team members [13]. Buthmann and Kleinert [14] stated that the key success factors for an effective six sigma coaching process include 1) Putting enough time in, 2) Contracting between the belt and coach, 3) Establishing a trustful relationship, 4) Being flexible, 5) Listening intently, among others.

Sustainability plays a vital role in implementing LSS, Rathi et al. (2022) focus on identifying and analyzing success factors for Green Lean Six Sigma (GLSS) implementation in Indian healthcare facilities, finding that “commitment of management” and “financial availability” are critical success factors while “embedding sustainable measures at each stage of the service,” “real-time data collection capability and effectiveness,” and “feedback and corrective actions” directly support GLSS implementation [a]. Moreover, Kaswan et al. (2022) propose a unified framework for integrating GLSS and Industry 4.0 to enhance organizational sustainability, providing systematic guidelines for project selection to solution sustainment [b]. Yadav et al. (2021) identify and prioritizes GLS performance improvement barriers. Management and financial constraints are the most critical, potentially aiding researchers and practitioners in implementing GLS more comprehensively [c]. Overall, these studies highlight the importance of adopting and implementing sustainable manufacturing methods, such as GLSS, to improve environmental and financial performance and sustain organizational viability.

The current research investigated studies about coaching; however, there is scarce literature focusing on LSS in general. Moreover, several studies only focused on the effectiveness of managerial coaching on employee behavior [15]. The results showed that managerial coaching is positively correlated with thriving at work. In addition, Rojon et al. [16] did a study only focusing on workplace coaching. The results showed that personal recommendations and client feedback were the two most essential coaching attributes. Given these, the study wanted to answer the research questions about employee preferences for LSS coaching using a Conjoint Analysis Approach. The research needs to fill this gap to cater to preference when it comes to LSS is a must to encourage more participants and would enhance the skills which can be applied in businesses. Therefore, to address this gap, the current research employs the Conjoint Analysis approach for analyzing employee preferences in terms of LSS coaching.

Conjoint analysis is a technique developed in the 1960s that is used to examine individuals' preferences in decision-making [17,18], mostly in marketing, education, leadership, and entrepreneurship. This allows researchers to understand individuals’ preferences based on ratings or rankings for products or offerings [16,19]. It is important to understand employee preferences on LSS coaching to contribute to the success of the implementation of Six Sigma projects across multiple industries and to successfully align companies to Industry 4.0.

The purpose of this study was to determine the preference of the employees completing different types of LSS projects on the coaching offered to them. Specifically, this study focused on employees completing any of the following LSS projects: Quick Win, Yellow Belt, Green Belt, and Black Belt. This is considered the first study that covered LSS coaching preference among different employees. The findings of this study will be beneficial to companies implementing LSS programs regarding employee preferences on LSS coaching. In addition, the results of this study will help companies implement and sustain better employee-oriented LSS programs, therefore driving higher rates of employee participation.

In the specific organization to which the research was conducted, a team of trained Green Belts and Black Belts serves as coaches to other employees engaged in their LSS projects. Coaching, therefore, represented the primary job function and area of responsibility for these Green Belts and Black Belts. Thus, it is essential to focus on the approach adopted by these coaches in providing support to their fellow employees. By examining the techniques and methodologies employed by the coaches, a comprehensive understanding may be obtained regarding.

By conducting an examination of the strategies, techniques, and methodologies employed by these coaches, a comprehensive understanding can be obtained regarding the effective delivery of coaching. This understanding can shed light on how coaching positively influences employee performance and contributes to achieving successful outcomes in LSS projects.

This paper is organized as follows: Section 1 covers the introduction and Section 2 represents the methodology which explained the participants, demographics, conjoint design, and statistical analysis. Section 3 presents the conjoint results for employees completing four different LSS projects: Quick Win, Yellow Belt, Green Belt, and Black Belt. Section 4 covers the discussion of the findings. Lastly, Section 5 covers the conclusion and recommendation.

## 2 Materials and methods

The primary purpose of the current research is to determine the preference of the employees completing different types of LSS projects on the coaching offered to them. Next, employees who have been trained with the Six Sigma methodology were asked to answer a survey questionnaire using a purposive sampling data collection technique. Twenty combined attributes about Six Sigma coaching preference were formulated using the conjoint design. Finally, Conjoint Analysis was utilized in analyzing the data using SPSS to understand individuals’ preferences based on ratings.

### 2.1 Participants

Prior to data collection, discussions were held with the management of the organization being studied. They recommended a target of gathering responses from approximately 25% of the organization's employees, which coincided with the number of employees who had undergone LSS Methodology training, specifically through a LSS Yellow Belt training session.

The study utilized a purposive sampling data collection technique due to the limited number of employees who have been trained with the Six Sigma Methodology [20]. A total of 100 employees participated in answering the 20 combined attributes about Six Sigma coaching preference. Ong et al. [17] suggested that the online distribution of surveys was sufficient when performing a conjoint analysis. Therefore, this study utilized MS Forms and distributed the online survey through e-mail and MS Teams to get the responses from the employees. Employees were informed through their respective LSS Coaches that the survey is part of the feedback collection initiative to further improve the LSS Coaching services.

### 2.2 Demographics

Table 1 presents the demographics of the study. The study tried to gather responses from 25% of the company's population or equivalent to 148 employees, however, was only able to collect 100 responses. The organization deemed this sample size sufficient for the purposes of the study, considering the limited availability of employees trained in LSS Methodology. Among the 100 respondents, 57 were completing a Quick Win project, 27 were completing a Yellow Belt project, 10 were completing a Green Belt project, and 6 were completing a Black Belt project. Due to lesser employees trained and exposed to Green Belt and Black Belt, most of the respondents were completing Quick Win and Yellow Belt projects. As the level of complexity of the project increases, fewer employees are available since completing Six Sigma projects follows a hierarchy [21]. This study was approved by Mapua University Research Ethics Committees. Informed consent was obtained from all participants prior to the data collection.

**Table 1:** Demographics (N: 100 respondents).

Lean Six Sigma Project	Job Level	N
Quick Win	Staff	50
	Team Lead	7
	Manager	0
	Executive	0
Yellow Belt	Staff	22
	Team Lead	4
	Manager	1
	Executive	0
Green Belt	Staff	6
	Team Lead	3
	Manager	1
	Executive	0
Black Belt	Staff	0
	Team Lead	3
	Manager	2
	Executive	1

### 2.3 Conjoint design

Table 2 presents the attributes of Six Sigma coaching. The 6 attributes considered were: Coaching Style, Frequency of Coaching Sessions, Duration of Coaching Sessions, Turn-around Time of Feedback, Documentation Review, and Mock Defense. To which, transactional, Laissez-Faire, and Democratic levels were considered for Coaching Style. Following is the frequency of Coaching Sessions weekly, Biweekly, and Monthly. Under Duration of Coaching Sessions, 30 min, 1 h, and 1 h and 30 min were considered with 1 week, 2 weeks, and 1 month as the Turn-around Time of Feedback. Lastly, both the Documentation Review and Mock Defense considered Yes and No levels.

**Table 2:** Attributes of lean six sigma coaching.

Attributes	Level
Coaching Style	Transactional
	Laissez-Faire
	Democratic
Frequency of Coaching Sessions	Weekly
	Biweekly
	Monthly
Duration of Coaching Sessions	30 min
	1 h
	1 h 30 min
Turn-around Time of Feedback	1 week
	2 weeks
	1 month
Documentation Review	Yes
	No
Mock Defense	Yes
	No

The first attribute considered was the coaching style. Lonczak [22] stated that matching the coaching style to a client's needs is essential for client success. Among the 10 most popular coaching styles were Transactional, Laissez-Faire, and Democratic. Transactional, as defined [23] is when the coach is implementing a task-driven and time-limited style aimed at performance enhancement. Laissez-Faire, as defined [24] is when the coach acts as a consultant and holds the client responsible as the primary process owner in driving the direction. Democratic, as defined [25] is when the coach includes the client to take an active role in the decision-making process, yet the coach still has the last word. The coaching-style can be agreed upon during the contracting stage [14].

The second attribute was the frequency of coaching sessions. Webb [26] stated that to increase the effectiveness of coaching sessions, one must consider the frequency of the sessions. The most common frequencies are weekly, biweekly, and monthly. Moreover, the consideration of the duration of coaching sessions was considered the third attribute. Webb [26] stated that from a survey conducted, most coaching sessions were conducted between 30 and 39 min, the second most popular one is between 60 and 69 min, and the third is more than 70 min. For the duration of coaching sessions, 30 min, 1 h, and 1 h 30 min were considered since these are widely used as standard meeting durations.

The fourth attribute was the turn-around time of feedback. Karna et al. [27] discussed that project feedback is a vehicle for learning in the organization. With well-timed feedback, problems are prevented and quick problem solving is enabled. For the turn-around time of feedback, 1 week, 2 weeks, and 1 month are considered. In addition, the fifth attribute was documentation review and whether employees would prefer it (yes) or not (no). Alby [28] stated that documentation reviews are structured ways to review the project's documentation. Furthermore, in the said reviews, it is crucial to know about the consistency and quality of information stated in the project. As LSS projects are data-driven [29], it is helpful to include this attribute.

The sixth attribute was mock defense and whether employees would prefer it (yes) or not (no). This attribute was considered as Sheridan [30] explained that the belts present their projects to team members, sponsors, and top management.

### 2.4 Statistical analysis

The SPSS 26 was used to generate the orthogonal design using conjoint analysis. A total of 20 stimuli were generated by the SPSS to measure the attributes for employee preference [17]. Table 3 presents the 20 stimuli evaluated by a 7-point Likert scale ranging from 1 as “strongly disagree” to 7 as “strongly agree.” A 7-point Likert scale is used to reflect the respondent's true evaluation more accurately [19,31]. The process for conjoint analysis involved the initiation process of developing the questionnaire through brainstorming, a review of the literature to come up with the attributes and levels, and a preliminary run.

**Table 3:** Stimulus.

Combination	Coaching Style	Frequency of Coaching Sessions	Duration of Coaching Sessions	Turn-around Time of Feedback	Documentation Review	Mock Defense
1	Transactional	Weekly	30 min	1 week	Yes	Yes
2	Transactional	Weekly	30 min	1 week	No	No
3	Laissez-Faire	Monthly	30 min	1 week	Yes	Yes
4	Laissez-Faire	Biweekly	30 min	2 weeks	No	No
5	Democratic	Weekly	1 h	2 weeks	Yes	Yes
6	Democratic	Weekly	1 h 30 min	1 week	No	Yes
7	Transactional	Biweekly	1 h 30 min	1 month	Yes	Yes
8	Transactional	Biweekly	1 h	1 week	Yes	No
9	Laissez-Faire	Monthly	1 h 30 min	1 week	Yes	No
10	Laissez-Faire	Biweekly	1 h	1 month	No	No
11	Democratic	Monthly	30 min	1 month	Yes	Yes
12	Democratic	Biweekly	30 min	1 week	No	No
13	Transactional	Weekly	30 min	2 weeks	Yes	No
14	Transactional	Monthly	1 h 30 min	2 weeks	No	No
15	Laissez-Faire	Monthly	30 min	1 month	Yes	No
16	Laissez-Faire	Biweekly	30 min	2 weeks	Yes	No
17	Democratic	Weekly	30 min	1 week	Yes	Yes
18	Democratic	Biweekly	30 min	2 weeks	Yes	No
19	Transactional	Weekly	30 min	2 weeks	No	Yes
20	Transactional	Biweekly	1 h	1 week	Yes	Yes

A total of 20 combinations were generated by the SPSS 26. To which, a preliminary run was conducted among 30 respondents. A Pearson's R result presented a 0.835 value. This means that the combinations have internal validity and could be utilized for data gathering [32]. Thus, the final form was created and distributed for evaluation among respondents.

## 3 Results

### 3.1 Quick Win projects

Table 4 presents the utilities and the average score of importance among the Six Sigma coaching attributes. This section specifically covers results for 57 employees completing their Quick Win projects. Higher utility values indicate a greater preference towards a certain level of the attribute. Moreover, a higher average score of importance reflects a higher preference for an attribute [19].

**Table 4:** Utilities and importance score for quick win projects.

Attributes	Preference	Utility Estimates	Std. Error	Average Score of Importance
Coaching Style	Transactional	0.076	0.151 0.184 0.161	13.494
	Laissez-Faire	−0.221		
	Democratic	0.145		
Frequency of Coaching Sessions	Weekly	0.138	0.198	11.589
	Biweekly	0.040	0.161	
	Monthly	−0.177	0.203	
Duration of Coaching Sessions	30 min	−0.002	0.149	10.633
	1 h	0.146	0.198	
	1 h 30 min	−0.143	0.197	
Turn-around Time of Feedback	1 week	0.071	0.144	4.662
	2 weeks	−0.015	0.162	
	1 month	−0.056	0.191	
Documentation Review	Yes	0.403	0.118	29.642
	No	−0.403	0.118	
Mock Defense	Yes	0.407	0.131	29.98
	No	−0.407	0.131	
Constant		3.745	0.131	

Table 5 presents the correlation results from the employees completing their Quick Win Projects. The value of Pearson's R is 0.922 and the value of Kendall's Tau is 0.815. The results being close to 1.00 indicate that there is a strong relationship between the observed and estimated preferences and that the orthogonal design is acceptable [32].

**Table 5:** Correlation from quick win projects.

	Value	Significance
Pearson's R	0.922	<.001
Kendall's Tau	0.815	<.001

Based on the result, the highest average score of importance was the mock defense (29.98%) with a preference to conduct it within the duration of the project. The second highest was the documentation review (29.64%) with a preference to have their coach conduct it within the duration of the project. The third highest was the coaching style (13.49%) with democratic as the choice of level. The fourth attribute was the frequency of coaching sessions (11.59%) with weekly as the choice of level. The fifth attribute was the duration of coaching sessions (10.63%) with 1 h as the choice of level. The last attribute was the turn-around time of feedback (4.66%) with 1 week as the choice of level.

Taking into consideration the levels considered by Quick Win Projects, they would consider Democratic, a weekly coaching session with a 1-h duration, and a 1-week feedback turnaround time with documentation review and the mock defense giving a value of 1.31. The least preferred combination includes Laissez-Faire, a monthly coaching session of 1 h and 30 min duration, a 1-month feedback turnaround time with no documentation and mock defense, which gives a value of −1.407.

### 3.2 Yellow Belt projects

Table 6 presents the utilities and the average score of importance for employees completing their Yellow Belt projects. This section specifically covers results for 27 employees that are completing the Yellow Belt project. Moreover, Table 7 presents the correlation results with Pearson's R-value of 0.926 and Kendall's Tau of 0.844. The results are considered to be acceptable [32].

**Table 6:** Utilities and importance score for yellow belt projects.

Attributes	Preference	Utility Estimates	Std. Error	Average Score of Importance
Coaching Style	Transactional	−0.079	0.143	12.824
	Laissez-Faire	−0.153	0.174	
	Democratic	0.232	0.153	
Frequency of Coaching Sessions	Weekly	0.248	0.188	14.716
	Biweekly	−0.054	0.153	
	Monthly	−0.194	0.193	
Duration of Coaching Sessions	30 min	−0.130	0.141	13.608
	1 h	0.269	0.188	
	1 h 30 min	−0.139	0.187	
Turn-around Time of Feedback	1 week	0.056	0.137	10.212
	2 weeks	0.125	0.154	
	1 month	−0.181	0.181	
Documentation Review	Yes	0.365	0.112	24.305
	No	−0.365	0.112	
Mock Defense	Yes	0.365	0.124	24.335
	No	−0.365	0.124	
Constant		3.763	0.124

**Table 7:** Correlation from yellow belt projects.

	Value	Significance
Pearson's R	0.926	<.001
Kendall's Tau	0.844	<.001

Based on the result, the highest average score of importance was the mock defense (24.34%) with a preference to conduct it within the duration of the project. The second highest was the documentation review (24.31%) with a preference to have their coach conduct it within the duration of the project. The third highest was the frequency of coaching sessions (14.72%) with weekly as the choice of level. The fourth attribute was the duration of coaching sessions (13.61%) with 1 h as the choice of level. The fifth attribute was the coaching style (12.82%) with democratic as the choice of level. The last attribute was the turn-around time of feedback (10.21%) with 2 weeks as the choice of level.

Considering the results of the Yellow Belt project, people would highly consider democratic, weekly coaching session around 1 h, 1–2 weeks feedback turnaround time, with documentation and mock defense resulting to 1.604 value. The least preferred would be Laissez-Faire, monthly coaching session, 30 min or 1 h and 30 min duration, 1-month feedback turnaround time, no documentation and mock defense resulting in −1.397 value.

### 3.3 Green Belt projects

Table 8 presents the utilities and the average score of importance for employees completing their Green Belt projects. This section specifically covers results for 10 employees finishing Green Belt projects. Table 9 presents the correlation results from the employees completing their Green Belt Projects. The value of Pearson's R is 0.853 and the value of Kendall's Tau is 0.731. The results is considered acceptable [32].

**Table 8:** Utilities and importance score for green belt projects.

Attributes	Preference	Utility Estimates	Std. Error	Average Score of Importance
Coaching Style	Transactional	0.246	0.211	20.075
	Laissez-Faire	−0.325	0.256	
	Democratic	0.079	0.225	
Frequency of Coaching Sessions	Weekly	0.087	0.276	7.988
	Biweekly	0.053	0.225	
	Monthly	−0.140	0.283	
Duration of Coaching Sessions	30 min	0.154	0.208	15.397
	1 h	0.130	0.277	
	1 h 30 min	−0.284	0.275	
Turn-around Time of Feedback	1 week	0.138	0.201 0.226 0.267	7.335
	2 weeks	−0.067		
	1 month	0.071		
Documentation Review	Yes	0.480	0.165 0.165	33.724
	No	−0.480		
Mock Defense	Yes	0.220	0.182 0.182	15.481
	No	−0.220		
Constant		3.390	0.183

**Table 9:** Correlation from green belt projects.

	Value	Significance
Pearson's R	0.853	<.001
Kendall's Tau	0.731	<.001

Based on the result, the highest average score of importance was the documentation review (33.72%) with a preference to conduct it within the duration of the project. The second highest was the coaching style (20.08%) with transactional as the choice of level. The third highest was the mock defense (15.48%) with a preference to have their coach conduct it within the duration of the project. The fourth attribute was the duration of coaching sessions (15.40%) with 30 min as the choice of level. The fifth attribute was the frequency of coaching sessions (7.99%) with weekly as the choice of level. The last attribute was the turn-around time of feedback (7.34%) with 1 week as the choice of level. The preferred levels resulted in 1.325.

Considering the least preferred levels, the combinations considered were Laissez-Faire, monthly frequency of coaching session, 1 h and 30 min coaching session, 2 weeks turnaround time of feedback, no documentation review, and no mock defense. This would lead to a −1.516 score value.

### 3.4 Black Belt projects

Table 10 presents the utilities and the average score of importance for employees completing their Black Belt projects. This section specifically covers results for 6 employees. In addition, Table 11 presents the correlation results from the employees completing their Black Belt projects. The value of Pearson's R is 0.926 and the value of Kendall's Tau is 0.764. The results being close to 1.00, a threshold of 0.70 indicate that there is a strong relationship between the observed and estimated preferences [32,33]. This indicates that the result is acceptable [32].

**Table 10:** Utilities and importance score for black belt projects.

Attributes	Preference	Utility Estimates	Std. Error	Average Score of Importance
Coaching Style	Transactional	0.014	0.243	5.083
	Laissez-Faire	−0.131	0.296	
	Democratic	0.117	0.259	
Frequency of Coaching Sessions	Weekly	0.260	0.318	14.286
	Biweekly	0.175	0.26	
	Monthly	−0.435	0.327	
Duration of Coaching Sessions	30 min	−0.228	0.24	7.568
	1 h	0.140	0.319	
	1 h 30 min	0.087	0.318	
Turn-around Time of Feedback	1 week	0.306	0.232	14.627
	2 weeks	0.099	0.261	
	1 month	−0.405	0.308	
Documentation Review	Yes	0.783 −0.783	0.191	32.199
	No		0.191	
Mock Defense	Yes	0.638	0.211	26.237
	No	−0.638	0.211	
Constant		3.233	0.211

**Table 11:** Correlation from black belt projects.

	Value	Significance
Pearson's R	0.926	<.001
Kendall's Tau	0.764	<.001

Based on the result, the highest average score of importance was the documentation review (32.20%) with a preference to have their coach conduct it within the duration of the project. The second highest was the mock defense (26.24%) with a preference to coach conduct it within the duration of the project. The third highest was the turn-around time of feedback (14.63%) with 1 week as the choice of level. The fourth attribute was the frequency of coaching sessions (14.29%) with weekly as the choice of level. The fifth attribute was the duration of coaching sessions (7.57%) with 1 h as the choice of level. The last attribute was the coaching style (5.08%) with democratic as the choice of level. This results in a 2.244 value for the preferred combination.

For the least preferred combination, Laissez-Faire was for coaching style, monthly frequency of coaching sessions, 30 min duration of coaching session, 1-month turnaround time for feedback, no documentation review, and no mock defense. This presented a value of 2.620.

## 4 Discussion

Table 12 represents the comparisons between the four LSS projects. The different attributes were ranked according to the perceived preference of employees currently taking one of the LSS projects. The first rank was the highest perceived importance, while the sixth rank was the lowest score of perceived importance.

**Table 12:** Comparison between four LSS projects.

Rank	Quick Win Projects	Yellow Belt Projects	Green Belt Projects	Black Belt Projects
1st	Mock Defense (Required)	Mock Defense (Required)	Documentation Review (Required)	Documentation Review (Required)
2nd	Documentation Review (Required)	Documentation Review (Required)	Coaching Style (Transactional)	Mock Defense (Required)
3rd	Coaching Style (Democratic)	Frequency of Coaching Sessions (Weekly)	Mock Defense (Required)	Turn-around Time of Feedback (1 week)
4th	Frequency of Coaching Sessions (Weekly)	Duration of Coaching Sessions (1 h)	Duration of Coaching Sessions (30 min)	Frequency of Coaching Sessions (Biweekly)
5th	Duration of Coaching Sessions (1 h)	Coaching Style (Democratic)	Frequency of Coaching Sessions (Weekly)	Duration of Coaching Sessions (1 h)
6th	Turn-around Time of Feedback (1 week)	Turn-around Time of Feedback (2 weeks)	Turn-around Time of Feedback (1 week)	Coaching Style (Democratic)

Based on the results, there are similarities among the preferences of employees completing different LSS projects. Employees completing a Quick Win project (29.98%) and employees completing a Yellow Belt project (24.34%) both regarded having a mock defense conducted within the duration of their project as the most preferred attribute, whereas employees completing a Green Belt project (15.48%) and employees completing a Black Belt project (26.24%) regarded it as their third and second most preferred attribute, respectively. According to Lantsoght [34], getting ready for one's defense is like preparing in the dark as most are confused about the flow of the defense, role of the coach, and the possible outcomes. This presents that it is likely for Quick Win and Yellow Belt projects to focus on their final output presentation as part of the requirement to finish the project. Kumar et al. [35] explained how the success of the implementation of LSS would be in the execution and implementation. With that, it is crucial to test the knowledge and understanding through mock defenses. Therefore, it is helpful to practice the presentation with one's colleagues and coach to prepare for the defense [36,37].

Additionally, employees completing a Green Belt project (33.72%) and employees completing a Black Belt project (32.20%) both regarded having a documentation review within the duration of their project as the most preferred attribute, whereas employees completing a Quick Win project (29.64%) and employees completing a Yellow Belt project (24.31%) regarded it as their second most preferred attribute. As discussed by Lim [38], a requirement in managing LSS projects is to track the status and its required documentation as it goes through the DMAIC phases. Moreover, Gay [12] stated that when inexperienced Green Belts are not provided sufficient coaching and support, they most likely will make mistakes in implementing the DMAIC methodology. Manufacturing in United Kingdom as presented by Antony et al. [39] explained how there is a lack of resources and documentation to implement a proper business strategy using LSS. Thus, documentation reviews are crucial for the success of the LSS project.

Following the third-highest rank among employees completing Quick Win projects, coaching style with democratic as the highest preferred level (0.145) followed by transactional (0.076), and Laissez-Faire as the least preferred (−0.221). Jiménez et al. [40] stated that democratic coaching could improve and increase one's performance and self-confidence. This is also supported by the results of employees completing Yellow Belt projects (0.232) and employees completing Black Belt projects (0.117) with democratic as the highest preferred level of the coaching style attribute and Laissez-Faire being the least preferred. In addition, Gellis [41] explained how Laissez-Faire has a negative correlation with the success of implementing LSS. This justifies how people perceived the coaching style to be of high importance to know and understand LSS better.

The frequency of coaching sessions ranked fourth for employees completing Quick Win projects (11.59%) and employees completing Black Belt projects (14.29%), third for employees completing Yellow Belt projects (14.72%), and fifth for employees completing Green Belt projects (7.99%). Webb [26] mentioned that the positive effects of coaching were higher when sessions occurred every 1–2 weeks, which is supported by the results of 3 out of 4 groups preferring a weekly coaching schedule. Similarly, Antony and Karaminas [5] explained how coaching presents one of the important attributes among Black Belt LSS earners. It is also explained to be one of the important attributes to have a positive implementation of the learnings [5].

Duration of coaching sessions ranked fifth for employees completing Quick Win projects (10.63%) and employees completing Black belt projects (7.57%), fourth for employees completing Yellow Belt projects (13.61%), and employees completing Green Belt projects (15.40%). Robbins [42] mentioned that coaching sessions spanning from 30 to 60 min allow the coach and the client to explore topics deeply while providing time to accommodate questions during the discussion. This is supported by the results of 3 out of 4 groups preferring a 1-h-long coaching session. Too much time spent on coaching or less time contributes to low retention and understanding [5].

Turn-around Time of Feedback ranks last for employees completing Quick Win projects (4.67%), employees completing Yellow Belt projects (10.21%), employees Green Belt projects (7.34%), and third for employees completing Black Belt projects (14.63%). Karna and Junnonen [27] discussed that by well-timed feedback, problems are prevented and quick problem solving is enabled. Since feedback is typically given during coaching sessions, the attributes of frequency of coaching session somehow cover the turn-around time of feedback attribute, thus having this as the lowest attribute of importance.

It can be deduced that employees, regardless of the type of LSS project they are currently completing, would prefer to have a mock defense and a documentation review conducted within the duration of their projects. This goes to show that employees value the quality of the output they produce and are concerned with implementing the LSS methodology correctly. Moreover, most of the employees have a similar preference when it comes to coaching style, frequency of coaching sessions as well as the duration of coaching sessions. Quick feedback, enough sessions for coaching, and democratic coaching were seen to be highly preferred and are understood to present better output and implementation of LSS.

### 4.1 Theoretical and practical contribution

The study took place within a Shared Service Organization situated in the IT-BPM sector of the Philippines. Conducting Lean Six Sigma (LSS) projects within this organization is particularly advantageous, given the need to remain relevant and competitive in a market projected to contribute USD 29 Billion in revenue and generate over 100,000 job opportunities between 2021 and 2022 [d].

Given that the Philippines is recognized as one of the leading destinations for outsourcing globally, it is imperative for the organization to sustain its competitive edge. To achieve this, the organization places emphasis on ensuring the successful completion of LSS projects initiated by its employees. To facilitate this process, assigned coaches provide valuable coaching support to project teams, enhancing the likelihood of project success and enabling the organization to maintain its competitive position.

Considering that this study is the first study that determines the Lean Six Sigma coaching attributes, the findings of this study can be used as a foundation to determine the employees' preference in the coaching they receive while completing a Lean Six Sigma project. Moreover, this study can be a basis to enhance the company's LSS programs. The results can be applied to companies worldwide.

Based on the result of this study, the success of the LSS programs and employee engagement in relation to LSS can be further explored. The approach could be helpful to companies with current LSS programs and those companies planning to have LSS programs that are considering the employees' preference when it comes to LSS coaching. Applying the findings of Rodin and Beruvides [43], LSS are considered to be cost-effective and are effective in company implementation to improve the system. Moreover, it was added that significant gains are evident when LSS can be applied. Thus, the findings of this study can be considered to enhance LSS project enrollees.

The focus on conducting mock defense and documentation review during coaching sessions should be the priority of companies. From an engagement standpoint, companies may plan to enhance their LSS programs to better involve their employees and increase their satisfaction. This will also lead to testing the knowledge and understanding of the people finishing the LSS project.

As more employees are encouraged to complete their LSS projects, organizations gain competitive advantage by having solid processes in place, practitioners that know where, when, and how to collect data, and interpret data into sound decisions that will help propel the company forward in technological advancement.

### 4.2 Limitations and future research

Despite the practical contributions, there are several limitations of this study. First, due to the different training requirements of the LSS projects and the length of time the company is exposed to the LSS methodology, this study was only able to collect a small number of respondents. It is recommended to evaluate the orthogonal design with a higher number of respondents, and if possible, include other companies. Second, this study only focused on LSS projects under the Quick Win and the DMAIC type. Studies may also include other LSS project types such as Define, Measure, Analyze, Design, Validate (DMADV) or Design for Six Sigma (DFSS), Process Reengineering, and Process Management [8]. Moreover, clustering method may be utilized to categorize the different projects available using machine learning algorithm like K-Means or C-Means. Lastly, the study only focused on the preference of the employees and did not consider the effect of achieving preference, its relation to employee satisfaction, employee engagement, and sustainability of LSS programs in a company. Further studies may consider the following points along with the employees’ preferences [44], especially after obtaining the LSS certification.

The scope of the study was specifically aimed at investigating the alignment of employee preferences regarding coaching while engaged in their LSS Projects under Quick Win and DMAIC Methodology. It is important to note that the study did not encompass the evaluation of project completion or the sustainability of outcomes after project finalization. Considering this, the authors recommend that future research endeavors should also consider the broader aspects of LSS project completion and the sustainability of achieved results.

## 5 Conclusions

Organizations worldwide have implemented the Lean Six Sigma methodology because of its promising benefits that include an increase in market share, an increase in customer satisfaction, and improved performance of products and services. With coaching as one of the crucial factors of a successful implementation of LSS projects, there is a need to understand the preference of employees completing LSS projects[[45], [46], [47], [48]]. The purpose of this study was to determine the preference of the employees completing different types of LSS projects on the coaching offered to them. A conjoint analysis approach with an orthogonal design was utilized to determine the preference of the employees.

Six attributes were considered which are coaching style, frequency of coaching sessions, duration of coaching sessions, the turn-around time of feedback, documentation review, and mock defense. The study utilized a purposive sampling data collection technique due to the limited number of employees who have been trained with the Six Sigma Methodology. Specifically, the respondents included 57 employees completing a Quick Win project, 27 employees completing a Yellow Belt project, 10 employees completing a Green Belt project, and 6 employees completing a Black Belt project. A total of 100 employees participated in answering the 20 combined attributes about Six Sigma coaching preference that was distributed via e-mail and MS Teams.

The results showed that employees, regardless of the projects they are currently completing, considered conducting mock defense and a documentation review within the duration of the project as the two highest preferences. It can be assumed that employees prefer their coaches to check their output prior to fully completing and implementing the LSS project. Moreover, it can be said that the employees value the quality of the output they produce. Most of the groups also have similar preferences when it comes to coaching style (democratic), frequency of coaching sessions (weekly), and duration of coaching sessions (1 h).

With this, the results of this study can help match employee preference with respect to LSS coaching. Companies worldwide can refer to this study as a basis to enhance their current LSS programs. Further studies may also include other LSS project types such as Define, Measure, Analyze, Design, Validate (DMADV) or Design for Six Sigma (DFSS), Process Reengineering, and Process Management. Lastly, including the effect of achieving employee preference, its relation to employee satisfaction, employee engagement, and sustainability of LSS programs in a company can be considered in future research to develop a more employee-oriented LSS program. Being the first study that considered the Lean Six Sigma coaching attributes, the findings of this study can be used as a foundation to determine the employees’ preference in the coaching they receive while completing a Lean Six Sigma project worldwide [[49], [50], [51], [52], [53]].

## Production notes

### Author contribution statement

Anna Luisa C. Guevarra: Yogi Tri Prasetyo: Conceived and designed the experiments; Performed the experiments; Analyzed and interpreted the data; Contributed reagents, materials, analysis tools or data; Wrote the paper.

Ardvin Kester S. Ong: Analyzed and interpreted the data; Contributed reagents, materials, analysis tools or data; Wrote the paper.

Klint Allen Mariñas: Analyzed and interpreted the data; Wrote the paper.

### Data availability statement

Data will be made available on request.

### Additional information

No additional information is available for this paper.

## Funding statement

This research was funded by Mapúa University Directed Research for Innovation and Value Enhancement (DRIVE)

## Declaration of competing interest

The authors declare that they have no known competing financial interests or personal relationships that could have appeared to influence the work reported in this paper.
